# *α*-Mangostin Induces Apoptosis and Cell Cycle Arrest in Oral Squamous Cell Carcinoma Cell

**DOI:** 10.1155/2016/5352412

**Published:** 2016-07-12

**Authors:** Hyun-Ho Kwak, In-Ryoung Kim, Hye-Jin Kim, Bong-Soo Park, Su-Bin Yu

**Affiliations:** ^1^BK21 PLUS Project, School of Dentistry, Pusan National University, Busandaehak-ro, 49, Mulguem-eup, Yangsan-si, Gyeongsangnam-do 50612, Republic of Korea; ^2^Department of Oral Anatomy, School of Dentistry, Pusan National University, Busandaehak-ro, 49, Mulguem-eup, Yangsan-si, Gyeongsangnam-do 50612, Republic of Korea; ^3^Department of Dental Hygiene, Dong-Eui University, 176 Eomgwang-ro, Busanjin-gu, Busan 47340, Republic of Korea

## Abstract

Mangosteen has long been used as a traditional medicine and is known to have antibacterial, antioxidant, and anticancer effects. Although the effects of *α*-mangostin, a natural compound extracted from the pericarp of mangosteen, have been investigated in many studies, there is limited data on the effects of the compound in human oral squamous cell carcinoma (OSCC). In this study, *α*-mangostin was assessed as a potential anticancer agent against human OSCC cells. *α*-Mangostin inhibited cell proliferation and induced cell death in OSCC cells in a dose- and time-dependent manner with little to no effect on normal human PDLF cells. *α*-Mangostin treatment clearly showed apoptotic evidences such as nuclear fragmentation and accumulation of annexin V and PI-positive cells on OSCC cells. *α*-Mangostin treatment also caused the collapse of mitochondrial membrane potential and the translocation of cytochrome c from the mitochondria into the cytosol. The expressions of the mitochondria-related proteins were activated by *α*-mangostin. Treatment with *α*-mangostin also induced G1 phase arrest and downregulated cell cycle-related proteins (CDK/cyclin). Hence, *α*-mangostin specifically induces cell death and inhibits proliferation in OSCC cells via the intrinsic apoptosis pathway and cell cycle arrest at the G1 phase, suggesting that *α*-mangostin may be an effective agent for the treatment of OSCC.

## 1. Introduction

Oropharynx tumors (oral cancer) are caused by tobacco and alcohol consumption and high-risk human papillomavirus (HPV) infection. Oral squamous cell carcinoma (OSCC), which is the most common type of oral cancer and frequently arises from the mucosa of the oropharynx and oral cavity, is an increasing national health problem [1–3]. Despite advances in the diagnosis and treatment (chemotherapy, radiotherapy, and surgery) of oral cancer, the overall five-year survival rate has remained at ~60% over the past two decades [4, 5].

The tropical fruit mangosteen (*Garcinia mangostana* Linn.) grows in Southeast Asia, and the pericarp of the mangosteen contains a variety of phytochemicals that are used as traditional medicines for disease management [6]. One of the phytochemical groups in the mangosteen pericarp is the xanthones, which are compounds that are associated with various biological activities including cardioprotective, antioxidant, anti-inflammatory, antibacterial, antiallergy, and anticancer activities [7]. Of all the mangosteen pericarp-derived xanthones, *α*-mangostin is the most abundant and has been shown to have potent anticancer effects against various types of cancer [8–13].

Apoptosis (type I programmed cell death) is the regulated death program that functions to maintain cellular homeostasis and eliminate damaged cells [14, 15]. Apoptotic cells are known to exhibit various features including nuclear condensation, DNA fragmentation, membrane blebbing, and an increase in the permeability of cells [16]. *α*-Mangostin was previously shown to induce apoptosis via mitochondrial dysfunction in human leukemia cells [17], while another study showed that *α*-mangostin acts as an effective anticancer agent against pancreatic tumors* in vitro* and* in vivo* [18]. Other studies have demonstrated antiproliferative and apoptotic effects of *α*-mangostin in head and neck squamous carcinoma cells [19, 20]. The anticancer effects of *α*-mangostin in human OSCC and the molecular mechanisms underlying these effects are poorly understood.

Therefore, in the present study, we examined whether *α*-mangostin-induced cell death is intimately linked with apoptosis and cell cycle arrest in human OSCC.

## 2. Methods

### 2.1. Reagents and Cell Culture

Human OSCC cell lines (HSC-2, HSC-3, and HSC-4) were provided by professor Sung-Dae Cho in the Department of Oral Pathology, School of Dentistry, Chonbuk National University (Jeonju, Korea). Human periodontal ligament fibroblast (PDLF) was purchased from Lonza (Basel, Switzerland). All OSCC and PDLF cells were cultured in Minimum Essential Medium/Earle's Balanced Salt Solution (MEM/EBSS) and Dulbecco's Modified Eagle Medium: Nutrient Mixture F-12 (DMEM/F-12), respectively, containing 4 mM L-glutamine, 1.5 g/L sodium bicarbonate, 4.5 g/L glucose, and 1.0 mM sodium pyruvate supplemented with 10% fetal bovine serum (FBS) (Hyclone, Logan, UT) and 1% penicillin-streptomycin (WELGENE, Daegu, Korea). Cells were incubated at 37°C in a humidified 5% CO_2_/95% air incubator. *α*-Mangostin was purchased from Chromadex (Irvine, CA). MTT was purchased from Sigma (St. Louis, MO), and caspase-3, PARP, and p21^waf1/cip1^ antibodies were purchased from Cell Signaling Technology (Beverly, MA). Cytochrome c, Bak, caspase-9, and GAPDH antibodies were purchased from Santa Cruz Biotechnology (Santa Cruz, CA). Goat anti-rabbit IgG and goat anti-mouse IgG antibodies were obtained from Enzo life sciences (Farmingdale, NY). A stock solution of *α*-mangostin (20 mM) was prepared in dimethyl sulfoxide (DMSO; Duchefa, Haarlem, Netherlands) and stored at −20°C until use. The stock solution was diluted to the indicated concentrations with MEM/EBSS when needed. At 70–80% confluence, cells were treated with various concentrations of *α*-mangostin for the indicated time-periods. Cells grown in culture medium containing the equivalent amount of DMSO without *α*-mangostin served as controls. The final concentrations of DMSO in these experiments were 0.002–0.1% (v/v).

### 2.2. MTT Assay for Cell Viability

The viability of HSC-2, HSC-3, and HSC-4 cells was determined using an MTT assay. The OSCC cells were cultured in 96-well plates (1 × 10^4^ cells/well) and incubated in the presence of various concentrations of *α*-mangostin (0–10 *μ*M) for different time-periods. After the treatment was terminated, the culture medium was removed and 100 *μ*L MTT (500 mg/mL) was added to each well. The cells were incubated for 4 h at 37°C, after which the resulting formazan crystals were solubilized in DMSO (150 *μ*L/well) with constant shaking for 15 min. The colored solution was quantified at 620 nm using an ELISA reader (Tecan, Männedorf, Switzerland). Cell viability was calculated as a percentage relative to controls. The reported data were obtained from at least three independent experiments.

### 2.3. Hoechst Staining for Morphological Assessment of Apoptosis

Vehicle- and *α*-mangostin-treated cells were harvested and cytocentrifuged onto a glass slide. The cells were stained with 10 *μ*g/mL Hoechst 33342 (Sigma) for 10 minutes at 37°C in the dark before being washed with PBS. The slides were mounted with glycerol and the cells were examined under a fluorescence microscope (Carl Zeiss, Göettingen, Germany). The number of cells exhibiting a condensed or fragmented nucleus was determined from random sampling of 3 × 10^2^ cells per experiment by a blinded observer.

### 2.4. Flow Cytometry Analysis for Apoptosis, Mitochondrial Transmembrane Potential (ΔΨ*m*) Measurements, and Cell Cycle

To quantify apoptosis in the early, late stage and necrosis, cells were seeded in 60 mm dishes (3 × 10^5^ cells/dish), cultured with *α*-mangostin for 24 h, and harvested and processed for apoptosis assay using annexin V-FITC apoptosis detection kit (Enzo, Farmingdale, NY), following indicated protocol specified by the manufacturer and examined using a CYTOMICS FC500 flow cytometer system (Beckman Coulter, Porterville, CA). The data were analyzed using MultiCycle software that allows for simultaneous analyses for cell cycle parameters and apoptosis. The MMP (mitochondrial membrane potential) was measured using DiOC_6_ (Molecular Probes, Eugene, OR) dye. The cells were loaded with a final concentration of 1 *μ*M DiOC_6_ solution at 37°C for 30 min and then analyzed using flow cytometry.

### 2.5. Immunofluorescent Staining for Detection of Cytochrome c Translocation

The cells were plated on coverslips and treated with *α*-mangostin. After 24 h, the cells were stained with 50 nM MitoTracker Red at 37°C for 30 min. After washing twice with PBS, the cells were fixed with 4% paraformaldehyde (PFA) in PBS for 15 min and washed three times with PBS. After permeabilization with 0.1% Triton X-100 and blocking with 1% BSA in PBS for 30 min, the cells were incubated with primary antibodies in 1% BSA overnight at 4°C. After washing with PBS, cells were incubated with FITC-conjugated secondary antibodies in 1% BSA-PBS for 1 hour and rinsed with PBS. The cells were imaged using a confocal microscopy (Zeiss LSM 750 laser-scanning confocal microscope; Carl Zeiss, Göettingen, Germany).

### 2.6. Western Blot Analysis

Cells (2 × 10^6^) were washed twice with ice-cold PBS, resuspended in ice-cold lysis buffer (CST, Boston, MA), and incubated at 4°C for 1 h. The resulting lysates were centrifuged at 24 × 3.75 g for 30 min at 4°C. The protein concentrations of the lysates were determined using a Bradford protein assay (Bio-Rad, Richmond, CA). Lysates (25 *μ*g protein/lane) were resolved by 10% SDS (sodium dodecyl sulfate)/PAGE. The proteins were transferred to polyvinylidene fluoride (PVDF) membranes (Millipore, Billerica, MA) which were then blocked before being incubated with the appropriate primary antibodies. Immunostaining with secondary antibodies was detected using SuperSignal West Femto substrate (Pierce, Rockford, IL).

### 2.7. Statistical Analysis

Reported data represent mean ± SE. Statistical significances between groups were analyzed using one-way analysis of variance (ANOVA) and Dunnett's comparison. Differences with probability (*p*) values <0.05 were considered statistically significant.

## 3. Results

### 3.1. *α*-Mangostin Induces Apoptotic Cell Death in Human Oral Squamous Cell Carcinoma Cell Lines

**Figure 1:**
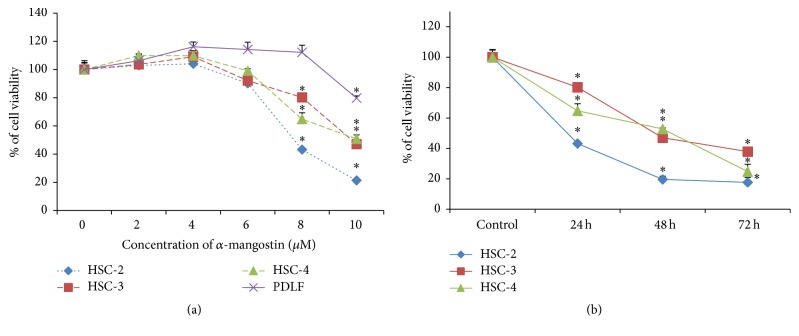
Effect of *α*-mangostin on cell viability in OSCC and PDLF cells. (a) OSCC and PDLF cells were treated with *α*-mangostin (0–10 *μ*M) for 24 h. (b) OSCC cells were treated with 8 *μ*M *α*-mangostin for 24 h–72 h. Data represent viability calculated relative to vehicle control-treated cells and are expressed as means ± SE from at least three experiments; ^*∗*^ *p* < 0.05 versus untreated cells.

**Figure 2:**
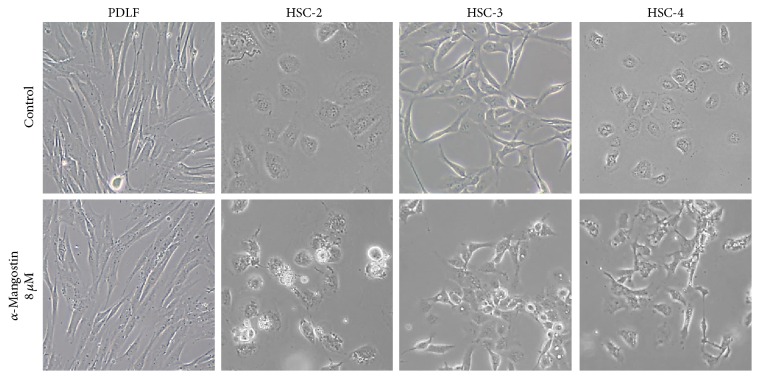
Morphological changes in *α*-mangostin-treated PDLF and OSCC cells. Cells were treated with *α*-mangostin for 24 h. Images were acquired with an optical microscope at 20x magnification.

To investigate the cytotoxic effect of *α*-mangostin, human OSCC cells (HSC-2, HSC-3, and HSC-4) and normal human periodontal ligament fibroblast (PDLF) cells were cultured with various concentrations of *α*-mangostin for 24 h. Cell viability was assessed using a MTT assay, which revealed that *α*-mangostin treatment resulted in a decrease in OSCC cell viability in a dose- and time-dependent manner without affecting normal PDLF cells (Figure 1). The IC_50_s of *α*-mangostin in OSCC cell types (HSC-2, HSC-3, and HSC-4) were 8–10 *μ*M (Figure 1(a)). *α*-Mangostin was also found to induce morphological changes such as membrane blebbing, cell shrinkage, and rounding in OSCC cells, but not in PDLF cells (Figure 2). These findings suggest that *α*-mangostin treatment induces cell death and morphological changes in OSCC cells.

**Figure 3:**
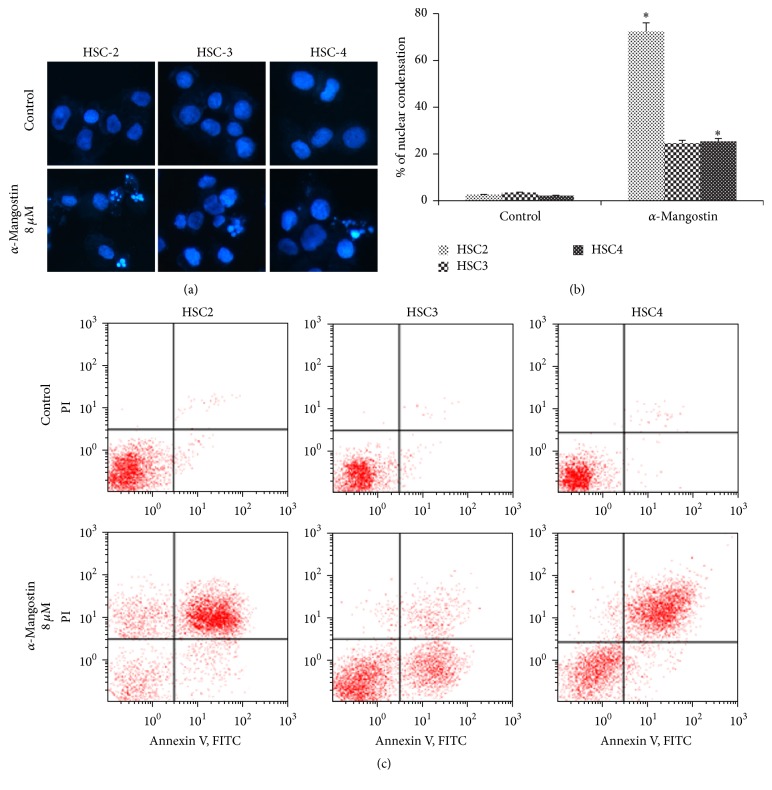
Induction of cell death in *α*-mangostin-treated OSCC cells via apoptosis. (a, b) After 24 h of treatment with *α*-mangostin, OSCC cells were stained with Hoechst and observed under a fluorescence microscope. (c) Analysis of apoptotic cells in untreated and *α*-mangostin-treated cells by annexin V and PI staining. Values represent mean ± SE of three independent experiments; ^*∗*^ *p* < 0.05 versus untreated cells.

To further investigate the biological mechanism underlying *α*-mangostin-induced cell death, cells were analyzed for the presence of apoptosis using Hoechst staining and flow cytometry. Following Hoechst staining, untreated cells exhibited round nuclei, while some *α*-mangostin-treated cells exhibited nuclear condensation and fragmentation. *α*-Mangostin had a significantly stronger effect in HSC-2 cells than in the other cell types (Figures 3(a) and 3(b)). To ascertain the dead ratio of OSCC cells, the cells were stained with annexin V and PI solution and analyzed by flow cytometry, and cells were treated with 8 *μ*M *α*-mangostin for 24 h. Compared with untreated cells, *α*-mangostin-treated cells exhibited significant increases in annexin V and PI-positive staining that indicated apoptosis (Figure 3(c)). Notably, the HSC-2 cells showed the highest apoptosis rate among the treated cells.

### 3.2. Mitochondrial Dysfunction and Caspase-Mediated Apoptosis Induced by *α*-Mangostin

**Figure 4:**
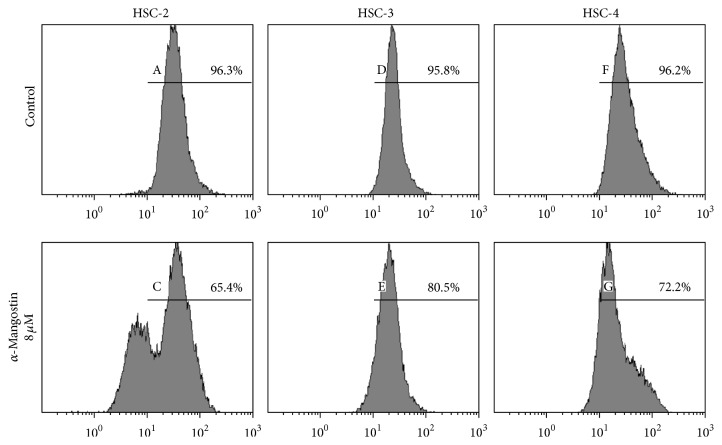
Collapse of mitochondrial membrane potential (ΔΨ*m*) in *α*-mangostin-induced apoptosis. Cells treated with 8 *μ*M *α*-mangostin for 24 h and incubated with 1 *μ*M DiOC_6_ were analyzed by flow cytometry.

**Figure 5:**
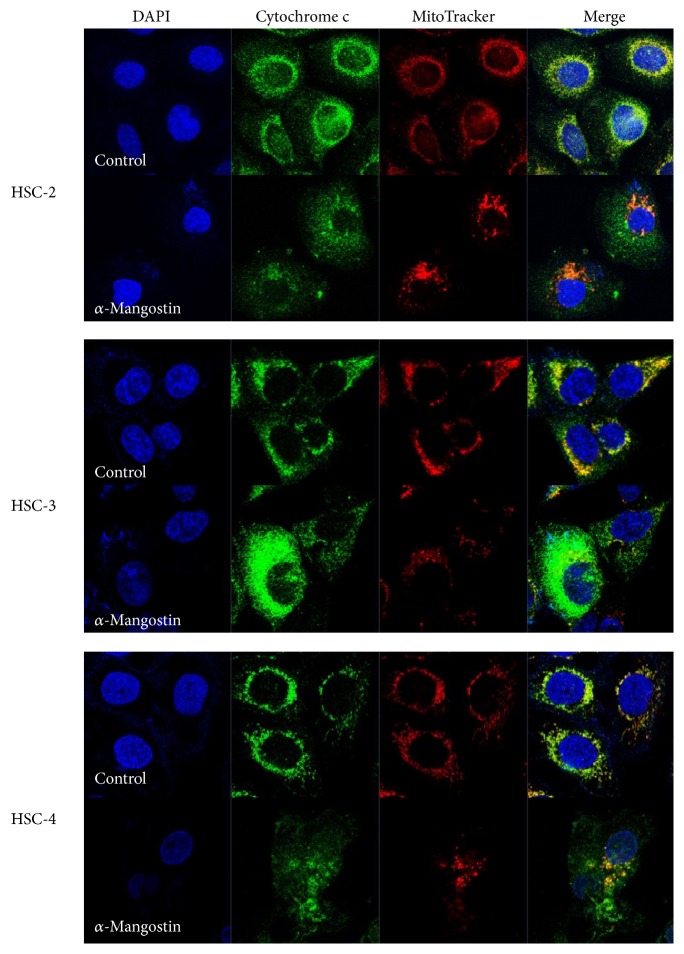
*α*-Mangostin-induced translocation of cytochrome c from mitochondria into the cytosolic fraction in OSCC cells. Cells were incubated with 8 *μ*M *α*-mangostin for 24 h before being stained with MitoTracker (red), cytochrome c (green), and DAPI (blue) to visualize mitochondria, cytochrome c, and nuclei, respectively. Images were acquired by confocal microscopy.

The intrinsic pathway of apoptosis is characterized by a key role for mitochondria, specifically by the dissipation of mitochondrial membrane potential (ΔΨ*m*), which is associated with mitochondrial dysfunction [21, 22]. Changes in ΔΨ*m* in *α*-mangostin-induced apoptosis were thus assessed using DiOC_6_. *α*-Mangostin was found to trigger a loss in ΔΨ*m* in the OSCC cell lines compared to the untreated controls (Figure 4). Loss in ΔΨ*m* may result in the release of cytochrome c into the cytosolic fraction and the translocation of cytochrome c was therefore subsequently assessed using immunofluorescent staining. As shown in Figure 5, treatment with 8 *μ*M *α*-mangostin induced the release of cytochrome c from the mitochondria into the cytosol in OSCC cells, whereas in the untreated cells, the cytochrome c staining colocalized with the MitoTracker signal.

**Figure 6:**
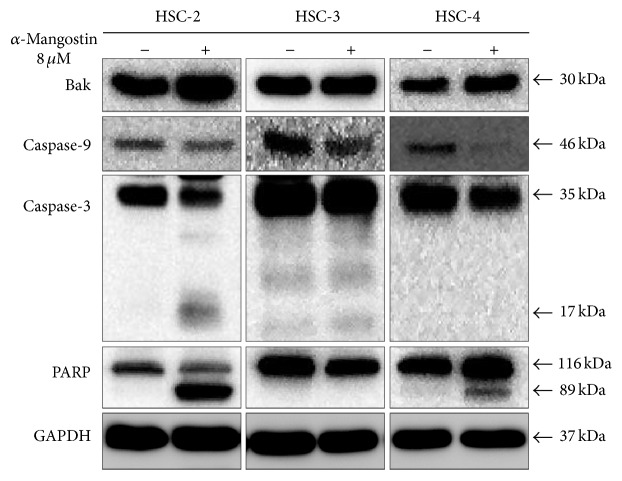
Activation of apoptosis-related proteins following *α*-mangostin treatment of OSCC cells. OSCC cells treated with 8 *μ*M *α*-mangostin were shown by western blotting to exhibit altered expression of apoptosis-associated proteins (Bak, caspase-9, caspase-3, and PARP).

To elucidate the molecular mechanisms underlying *α*-mangostin-induced apoptosis, the expression levels of apoptosis-associated proteins such as Bak, caspase-9, caspase-3, and PARP were assessed using western blot analysis. The protein expression levels of Bak, a proapoptotic Bcl-2 family member, were found to increase in response to *α*-mangostin, while the expression levels of caspase-9, caspase-3, and PARP (normalized to GAPDH expression) decreased. The expression levels of the cleaved forms of caspase-3 and PARP, in particular, were shown to increase significantly in HSC-2 cells (Figure 6). These results clearly demonstrate that *α*-mangostin-induced apoptosis involves the intrinsic apoptosis pathway and caspase-cascades.

### 3.3. *α*-Mangostin Regulates Cell Cycle Distribution through a G1 Phase Arrest

**Figure 7:**
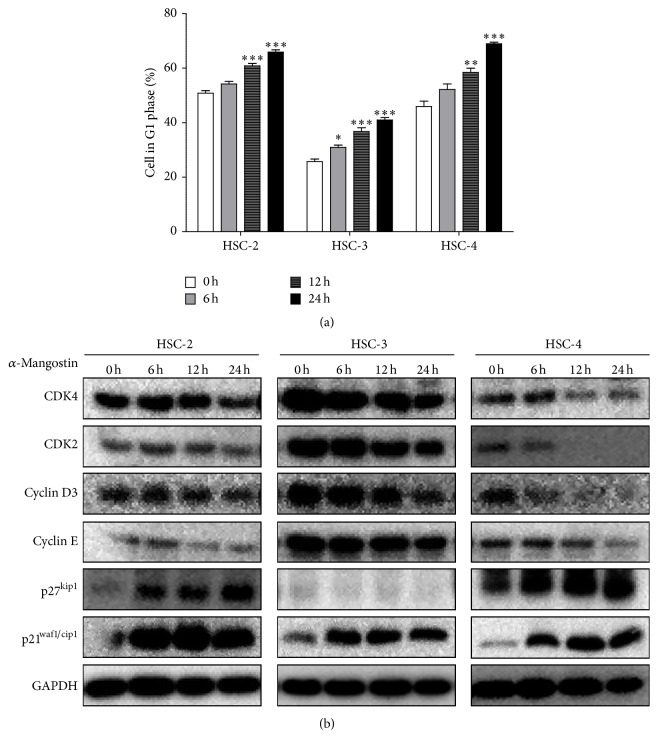
*α*-Mangostin arrests the cell cycle G1 phase in a time-dependent manner. Cells were treated with 8 *μ*M *α*-mangostin for 6, 12, and 24 h before being subjected to (a) cell cycle distribution analysis by flow cytometry and (b) western blotting to assess the expression levels of cell cycle-associated proteins (CDK4, CDK2, cyclin D3, cyclin E, p27^kip1^, and p21^waf1/cip1^) normalized to GAPDH expression. Values represent mean ± SE of three independent experiments; ^*∗*^ *p* < 0.05 versus untreated cells; ^*∗∗*^ *p* < 0.01 versus untreated cells; ^*∗∗∗*^ *p* < 0.001 versus untreated cells.

To assess whether *α*-mangostin arrests specific cell cycles, OSCC cells were treated with 8 *μ*M *α*-mangostin for 6, 12, and 24 h. Cell cycle population analysis was subsequently performed using flow cytometry after PI staining. The results of this analysis (Figure 7(a)) show that the cell distribution in the G1 phase became significantly stagnated in a time-dependent manner in the mangostin-treated group compared to the vehicle-treated group. The expression levels of cell cycle regulatory proteins including CDK4 (cyclin-dependent kinase-4), CDK2, cyclin D3, cyclin E, and p27^kip1^ were also assessed. Each CDK/cyclin complex is involved in the progression or transition of the cell cycle. Cyclin D interacts with CDK4 and cyclin E interacts with CDK2 to modulate cell cycle distribution in the G1 phase [23, 24].  Figure 7(b) shows that *α*-mangostin downregulated the expression of CDK4, CDK2, cyclin D3, and cyclin E, whereas p27^kip1^ and p21^waf1/cip1^, a CDK inhibitor, were upregulated by *α*-mangostin in a time-dependent manner. The p27^kip1^ protein, however, was not expressed in HSC-3 cells. These data reveal that *α*-mangostin leads to the arrest of the cell cycle at the G1 phase by inhibiting CDK/cyclin complex activities.

## 4. Discussion

The subsurface phytochemicals of the mangosteen pericarp include over 200 xanthones. The xanthones exhibit notable biological activities [7, 25] and among the many xanthone compounds, *α*-mangostin, *β*-mangostin, *γ*-mangostin, and gartanin are the most frequently researched [26]. Of these compounds, *α*-mangostin was shown to have the strongest apoptotic activity in human melanoma cells and, compared to other similar compounds, *α*-mangostin was shown to be the most effective agent in human cancer cell lines [27, 28]. *α*-Mangostin has also been reported to act as an anticancer agent against various types of cancer cells including canine osteosarcoma cells, human colorectal cancer cells, and pancreatic cancer cells [6, 29–32]. This suggests that *α*-mangostin may be considered a prospective anticancer agent; however, the biological mechanism underlying *α*-mangostin-induced cell death in human OSCC cell lines has not been well studied.

In the present study, we report that *α*-mangostin has antitumor effects against the human OSCC cell lines HSC-2, HSC-3, and HSC-4. The observed antitumor effects were accompanied by the inhibition of cell proliferation, the acceleration of mitochondria-controlled apoptosis, and cell cycle arrest with no toxicity in normal human PDLF cells. In our preliminary work, the cytotoxicities of *α*-mangostin and gartanin, both extracts of the mangosteen pericarp, were compared in human OSCC cells, and *α*-mangostin was found to have more potent cytotoxic effects than gartanin in OSCC cell lines. In these preliminary experiments, *α*-mangostin was also shown to inhibit cell viability in a dose- and time-dependent manner and to induce morphological changes in human OSCC cell lines but not in normal PDLF cells under the same conditions (Figures 1 and 2). Among the three human OSCC cell lines, HSC-2 cells were shown to be most sensitive to *α*-mangostin.

We furthermore clarified using Hoechst staining and flow cytometry with annexin V and PI staining that *α*-mangostin does induce apoptosis in human OSCC cells (Figure 3). Apoptosis is known to occur via two principal pathways: the extrinsic pathway is activated by death receptor stimulation, while the intrinsic pathway responds to DNA damage and other kinds of stimuli that are dominated by mitochondria [14, 33]. Mitochondria play a pivotal role in the apoptotic machinery, and when the intrinsic pathway is activated, a sequence of events is triggered. These events include the depolarization of the MMP, the release of cytochrome c from mitochondria into the cytoplasm, and significant activation of caspases involved in the intrinsic pathway [34]. A number of studies have demonstrated that *α*-mangostin induces apoptosis through the stimulation of mitochondrial functions [11, 12, 17]. In this study, we further demonstrated that *α*-mangostin also induces apoptosis in human OSCC cells via ΔΨ*m* depolarization and the release of cytochrome c, hallmarks of the mitochondrial pathway (Figures 4 and 5). The expressions of Bak, caspase, and PARP, which are considered critical regulators of apoptosis, were found to be activated by *α*-mangostin treatment (Figure 6), while Bcl-2 expression was not detected in the OSCC cells. In these experiments too, *α*-mangostin was found to have the most pronounced effect on HSC-2 cells compared with the other OSCC cells.

Cell cycle regulation is essential to the survival, proliferation, and differentiation of cells, and cell cycle dysregulation may lead to carcinogenesis [35, 36]. *α*-Mangostin-induced cell cycle arrest at the G1 phase has been observed in prostate cancer, human breast cancer, and colon cancer [8, 37, 38]. Similarly, in our study, *α*-mangostin treatment led to an accumulation of cells in the G1 phase accompanied by upregulation of the expression of p27^kip1^ and p21^waf1/cip1^, a known CDK inhibitor. The cyclin/CDK complexes (controllers of the cell cycle), however, were downregulated by *α*-mangostin treatment in a time-dependent manner (Figure 7).

The findings of the present study demonstrate that *α*-mangostin induces apoptosis in human OSCC cells and that this apoptotic cell death is accompanied by the dysregulation of mitochondrial function as well as cell cycle arrest. These findings provide evidence in support of *α*-mangostin as a novel, potential anticancer agent.

## 5. Conclusions

The present study described that *α*-mangostin could be a potential anticancer agent for human OSCC and provides valuable data for the development of a novel anticancer strategy.
